# Comparison of presentations to the emergency department during the COVID-19 pandemic (COPED-C)

**DOI:** 10.1093/pubmed/fdab059

**Published:** 2021-03-09

**Authors:** A Kociejowski, C Hobart, R Jina, I Aberman, E Backhurst, A Beaumont, J Crompton, R Sneep, F Cantle, H Dodhia

**Affiliations:** Health Intelligence Team, Public Health Directorate, Adults and Health, Lambeth Council, London SW2 1EG, UK; Health Intelligence Team, Public Health Directorate, Adults and Health, Lambeth Council, London SW2 1EG, UK; Emergency Department, King’s College Hospital NHS Foundation Trust London, London SE5 9RS, UK; GKT School of Medical Education, King’s College London, London WC2R 2LS, UK; GKT School of Medical Education, King’s College London, London WC2R 2LS, UK; GKT School of Medical Education, King’s College London, London WC2R 2LS, UK; Health Intelligence Team, Public Health Directorate, Adults and Health, Lambeth Council, London SW2 1EG, UK; Emergency Department, King’s College Hospital NHS Foundation Trust London, London SE5 9RS, UK; Emergency Department, King’s College Hospital NHS Foundation Trust London, London SE5 9RS, UK; Health Intelligence Team, Public Health Directorate, Adults and Health, Lambeth Council, London SW2 1EG, UK

**Keywords:** emergency care, health intelligence, public health

## Abstract

**Background:**

Concerns have been raised that patients requiring emergency care may not have accessed healthcare services during coronavirus disease 2019 (COVID-19) lockdown.

**Methods:**

This case control study aimed to understand changes in characteristics and diagnosis of patients attending a large UK Emergency Department (ED) during the first wave of the COVID-19 pandemic (March–May 2020) compared with equivalent weeks in 2019.

**Results:**

We found a 50.7% drop in first attendances to the ED in 2020. Likelihood of attendance and admission decreased for paediatric patients and increased for patients ≥ 46 years, and for men. Likelihood of admission increased for all Black ethnic groups and for patients from the most deprived index of multiple deprivation quintiles. This shift to an older, male, more deprived patient population with greater representation of ethnic minority groups was amplified in the ‘Infections’ diagnostic category.

**Conclusions:**

COVID-19 has dramatically impacted ED usage. Our analysis contributes to local resource planning and understanding of changes in healthcare-seeking behaviour during the pandemic. Future research to identify positive behaviour changes could help sustain a reduction in non-urgent visits in the longer term.

## Background

In response to the coronavirus disease 2019 (COVID-19) pandemic the UK government implemented several disease control measures, including a nationwide ‘lockdown’ on 23rd March 2020, restricting people to their homes other than for essential travel or exercise and closing all non-essential businesses. Emergency Department (ED) attendances significantly reduced subsequently, with the Royal College of Emergency Medicine (RCEM) reporting 89 584 ED attendances in the week following lockdown compared with 120 356 the previous week.^1^ March–May 2020 saw the lowest number of attendances reported since national data collection began in November 2010.^2–5^

Health professionals have voiced concerns that patients requiring emergency care may not have been accessing essential services during lockdown, resulting in higher morbidity and mortality compared with pre-COVID-19. The full impact of this is not yet fully understood but excess deaths (above the 5-year average) over and above those directly attributable to COVID-19 have been observed for the majority of weeks from week ending 27 March to week ending 15 May 2020.^6^ This includes an apparent shift in location of death, with more excess deaths occurring at home compared with hospital. Conversely, the reduction in ED attendances may represent appropriate choices to access other healthcare services or undertake self-care.^7^

National evidence demonstrates the shift in ED attendances during COVID-19 lockdown by age and diagnosis, but to our knowledge there is no analysis of local changes to ED attendance which considers changes in wider patient characteristics including ethnicity and deprivation.^8^

This comparative study aimed to describe and compare the characteristics and primary diagnosis of patients accessing King’s College Hospital ED before and during the first wave of the COVID-19 pandemic. The analysis aimed to improve understanding of changes in local health-seeking behaviours and thereby help local resource planning and development of local interventions to encourage ED attendance for those who need it while sustaining appropriate reductions in non-urgent visits. This will contribute to a new RCEM policy, ‘COVID-19: Resetting Emergency Department Care’, of which a fundamental aim is to ensure that EDs must not ever become overcrowded again.

## Methods

### Design

This study was a single centre retrospective case control study of all patients attending the ED at King’s College Hospital between 23 March and 15 May for 2020 and equivalent weeks in 2019.

### Setting

Kings College Hospital is a 950-bed university teaching hospital in South London. It primarily serves the London boroughs of Lambeth, Southwark, Lewisham and Bromley with a local population of over 1.2 million and is a tertiary referral centre for the South East and Kent region.

### Participants

First attendance records of all patients attending the ED were included for analysis. Repeat attendances were excluded. This period was selected to cover the start of the first COVID-19 ‘lockdown’ in the UK, to identify any potential association between the impact of COVID-19 in the UK and healthcare-seeking behaviour at the ED.

### Analysis

Variables included patient demographic characteristics (age, sex, ethnicity and index of multiple deprivation [IMD] derived from patient postcode of residence) and clinical categories (by condensed diagnosis and attendance outcome). See Supplementary Table 1 for groupings. Patients admitted from their attendance were analyzed as a proxy measure of severity.

**Table 1:** Study population characteristics: patients attending ED in 2020 and 2019 lockdown weeks (first attendances) (*n* = 24 503)

	*2020*		*2019*				
	*Number of first attendances*	*%*	*Number of first attendances*	*%*	*2019–2020% decrease*	*95% CI*	
Age (years)	Mean: 41.8 (95% CI 41.3–42.3)		Mean: 36.0 (95% CI 35.7–36.4)				
0–5	679	8.41	1951	11.88	65.20	63.04	67.31
6–18	548	6.78	2173	13.24	74.78	72.90	76.60
19–45	3324	41.10	6647	40.49	49.99	48.78	51.20
46–64	2130	26.34	3467	21.12	38.56	36.94	40.21
65–84	1098	13.58	1735	10.57	36.71	34.44	39.03
≥85	293	3.62	414	2.52	29.23	24.89	33.87
Missing data	15	0.19	29	0.18	48.28	29.45	67.47
Total	8087	100	16 387	100	50.65	49.88	51.42
Sex							
Female	4012	49.61	8456	51.51	52.55	51.48	53.62
Male	4060	50.20	7958	48.48	48.98	47.88	50.09
Missing data	15	0.19	2	0.01			
Total	8072	100	16 414	100	50.82	50.05	51.59
Ethnicity							
Asian	400	4.95	908	5.53	55.95	52.65	59.21
Black African	868	10.73	2013	12.26	56.88	54.68	59.06
Black Caribbean	630	7.79	1338	8.15	52.91	50.20	55.62
Black Other	831	10.28	1952	11.89	57.43	55.20	59.63
Latin American	144	1.78	292	1.78	50.68	44.80	56.56
Mixed ethnicity	290	3.59	591	3.60	50.93	46.82	55.03
Other ethnic group	573	7.09	1118	6.81	48.75	45.78	51.72
White British	2442	30.20	4958	30.20	50.75	49.34	52.15
White other	822	10.16	1742	10.61	52.81	50.44	55.18
Missing data	1087	13.44	1504	9.16	27.73	25.48	30.06
Total	8087	100	16 416	100	50.74	49.97	51.50
IMD quintile							
Most deprived 1	2020	24.98	4123	25.12	51.01	49.47	52.54
2	3372	41.70	6628	40.38	49.12	47.91	50.34
3	1841	22.76	3770	22.97	51.17	49.56	52.77
4	527	6.52	1180	7.19	55.34	52.45	58.20
Least deprived 5	180	2.23	393	2.39	54.20	49.13	59.20
Missing data	147	1.82	322	1.96	54.35	48.73	59.88
Total	7940	100	16 094	100	50.66	49.89	51.44

Anonymised data were extracted from the A&E software (Symphony) to Excel 2013.^9^ Initial descriptive analysis was performed in Excel 2013.^9^ Multivariate logistic regression analysis was conducted using STATA-14 controlling for changes seen in ED attendance, admission and primary diagnosis due to confounders of age, sex, ethnicity and IMD quintile.^10^ Data sharing was approved via the Information Governance team at King’s College hospital.

## Results

In total, 31 104 individual ED attendances were recorded across the study period. Of these, 24 503 first attendances were identified for descriptive analysis across both years during lockdown weeks (16 416 first attendances in 2019; 8087 in 2020); any repeat attendances were excluded. 24 017 patient records were included in the multivariate logistic analysis (486 records had missing data and were excluded).

## Descriptive analysis

### ED first attendances

Overall, there was a 50.7% drop in first attendances in 2020 compared with 2019 lockdown weeks (16 416 first attendances in 2019; 8087 in 2020).

The greatest drop in first attendances by age was seen in children aged 18 and under while the smallest drop was seen in the ≥85 year group.

Attendances approximately halved for both men and women, with a greater decrease seen among men. Attendances dropped by around half for all ethnic groups, with the largest drop in attendances in the Black African, Black Other and Asian ethnic groups. Attendances by deprivation dropped across all groups with a slightly larger drop seen in IMD quintile 4 (one of the least deprived quintiles). See Table 1 for detail.

### Admissions

Admissions decreased across all patient groups, with most decreases amplifying the changes seen in attendance. Younger patients (aged <  18 years) saw a far larger percentage reduction in admissions compared with patients aged 46 years and older. Although men and women experienced similar reductions in attendances, men had proportionally higher admissions compared with women. Changes seen in attendance by ethnicity were amplified in admissions data, with a much smaller decrease in admissions among all Black and Ethnic minority groups compared with White ethnic groups. While decreases in attendance by deprivation were similar across all deprivation quintiles, there was a marked change in admissions across deprivation quintiles, with patients from the least deprived quintiles experiencing the greatest percentage decrease in admissions. See Supplementary Table 2 for further detail.

### Diagnosis

ED attendances decreased across all diagnosis categories, with three exceptions: ‘general surgical emergencies’, ‘respiratory infections’ and ‘vulnerable person’ (homeless, safeguarding concerns etc.). Several diagnostic categories saw large (>60%) decreases. These included fractures and musculoskeletal; ophthalmology and ENT emergencies; gastrointestinal infections; gynaecology and obstetrics non-emergencies; other (administrative, prescribing and social attendances); central nervous system emergencies (excluding stroke) and respiratory (excluding infection). Smaller decreases (~30% decrease) were seen in clinically significant categories including cardiac and stroke. See Supplementary Table 3 for details.

Three condensed diagnosis categories accounting for the largest proportion of ED attendances in 2019 and 2020 were selected for further analysis—‘Injuries and trauma’; ‘Medical emergencies’; ‘Infections’.

### Multivariate logistic regression analysis

Multivariate logistic regression analysis compared attendances, admissions and diagnosis between 2019 and 2020 and adjusted for potential confounders of age, sex, ethnicity and deprivation (IMD quintile).

### ED attendances

Table 2 below shows the adjusted odds of ED attendances after adjusting for age, sex, ethnicity and IMD, comparing 2020 with 2019. In 2020 the odds of ED attendance were significantly lower for those aged 0–5 and 6–18 years and significantly higher for older patients aged ≥46 years.

**Table 2:** ED attendances—adjusted odds ratio (AOR) of attending ED during 2020 ‘lockdown’ weeks compared with 2019, by patient characteristics

	*AOR*	*95% CI (confidence interval)*		**P* value*
Age (years)				
19–45	Ref			
0–5	0.70	0.63	0.77	<0.0001
6–18	0.52	0.47	0.57	<0.0001
46–64	1.25	1.17	1.34	<0.0001
65–84	1.32	1.21	1.44	<0.0001
≥85	1.49	1.27	1.74	<0.0001
Sex				
Female	Ref			
Male	1.09	1.03	1.15	0.003
Ethnicity				
White British	Ref			
Asian	0.95	0.83	1.08	0.395
Black African	0.90	0.82	0.99	0.034
Black Caribbean	0.91	0.82	1.02	0.099
Black Other	0.95	0.86	1.05	0.317
Latin American	1.06	0.86	1.31	0.581
Mixed ethnicity	1.22	1.05	1.42	0.011
Other ethnic group	1.09	0.97	1.22	0.144
White other	1.03	0.94	1.14	0.519
Unknown/missing	1.49	1.36	1.64	<0.0001
IMD quintile				
Least deprived 5	Ref			
Most deprived 1	1.16	0.96	1.40	0.124
2	1.19	0.99	1.43	0.062
3	1.14	0.95	1.38	0.169
4	1.01	0.82	1.25	0.896

Men were slightly more likely to attend the ED than women in 2020.

Black African patients were less likely to attend the ED than White British patients in 2020 compared with 2019. Patients reporting Mixed ethnic background were more likely to attend in 2020.

No significant differences were seen in ED attendance by deprivation comparing patients attending in 2020 versus 2019.

### Admissions

Table 3 shows the adjusted odds for admissions in 2020 patients compared with 2019. Those aged 0–5 and 6–18 years were less likely to be admitted in 2020 compared with 2019. Those aged older than 46 years old were more likely to be admitted to hospital. Men were more likely to be admitted to hospital in 2020 compared with women.

**Table 3:** ED admissions—adjusted odds ratio (AOR) of requiring hospital admission during 2020 ‘lockdown’ weeks compared with 2019, by patient characteristics

	*AOR*	*95% CI*		**P* value*
Age (years)				
19–45	Ref			
0–5	0.60	0.45	0.82	<0.0001
6–18	0.56	0.42	0.77	<0.0001
46–64	1.70	1.44	2.01	<0.0001
65–84	1.92	1.62	2.27	<0.0001
≥ 85	1.85	1.48	2.31	<0.0001
Sex				
Female	Ref			
Male	1.28	1.14	1.45	<0.0001
Ethnicity				
White British	Ref			
Asian	1.27	0.96	1.68	0.091
Black African	1.58	1.29	1.93	<0.0001
Black Caribbean	1.34	1.09	1.66	0.006
Black other	1.50	1.20	1.88	<0.0001
Latin American	1.58	0.94	2.66	0.09
Mixed ethnicity	1.19	0.78	1.82	0.41
Other ethnic group	1.31	1.02	1.68	0.04
White other	1.31	1.03	1.67	0.03
Unknown	1.81	1.46	2.25	<0.0001
IMD quintile				
Least deprived 5	Ref			
Most deprived 1	1.79	1.24	2.58	0.002
2	1.72	1.20	2.47	0.003
3	1.62	1.11	2.34	0.011
4	1.27	0.84	1.91	0.253

The likelihood of admission increased for ‘Black African’, ‘Black Caribbean’ and ‘Black other’ groups as well as the ‘White other’ and ‘Unknown’ groups.

Admissions were progressively more likely in patients from the three most deprived quintiles (quintiles 1–3) compared with those from the least deprived quintile (quintile 5).

## Diagnosis

Three condensed diagnosis categories accounting for the largest proportion of ED attendances were analyzed: ‘Injuries and trauma’; ‘Medical emergencies’ and ‘Infections’. The most significant changes were seen in the ‘Infections’ category. Full details are available in Supplementary Table 4.

### Injuries and trauma

#### Attendances

ED attendance for injuries was more likely in 2020 in children aged 0–5 years; but half as likely in those aged 6–18 years. Other changes by age group lacked statistical strength. Male attendances with ‘Injuries’ were less likely than female attendances. Compared with the White British group, there was evidence of a decrease in attendances for the following ethnic groups –Asian; Black African; Black Caribbean. There was no statistical evidence for a change in patient attendance by IMD.

#### Admissions

Evidence showed a statistically significant decrease in admissions for the 6–18 years old group. There was no statistical evidence to support a significant change in admissions for ‘Injuries and Trauma’ by any other group including age, sex, ethnicity and IMD.

### Medical emergencies

#### Attendances

Medical emergencies were less likely in all children. There was evidence for an association between ethnicity and increased odds of attendance for medical emergency in two groups: ‘Black other’ and ‘White other’. There was no statistical evidence to support a significant change in attendance across other variables.

#### Admissions

Admissions were less likely in children aged 6–18 years and increased for patients aged 65–84. Likelihood of admission increased for ‘Black African’ and ‘Black Other’ ethnic groups and patients from the most deprived IMD quintile. Other changes observed lacked statistical support to infer significance.

### Infections

#### Attendances

Younger age groups were less likely to attend with ‘Infections’ in 2020. Likelihood of attendance increased for all groups aged ≥ 46 years. Males were more likely to attend than females in 2020.

#### Admissions

Changes seen in ED attendances for infection were amplified in admissions data. Admissions were less likely for children aged 0–5 years with no change in the 6–18 year group. Likelihood of admission increased for all groups aged ≥ 46 years. Males were more likely to require admission than females. Admission was more likely across several minority ethnic groups: Asian; Black African; Black Caribbean. There was insufficient evidence to infer a significant change in admissions by deprivation quintile.

## Discussion

### Main findings of this study

We found a significant drop in the number of ED attendances during 2020 compared with 2019, particularly during lockdown weeks. Reductions in attendances occurred across every demographic characteristic examined (age, ethnicity, gender and deprivation). However, there were differences in the extent to which attendances dropped across some of these characteristics.

The most significant shift in attendance demographics was by age, with proportionally fewer attendances among 0–18 year olds and more among the older population.

During lockdown, children (except those identified as ‘vulnerable’ or whose parents were keyworkers) could not attend childcare or education settings. This, combined with wider social distancing measures, probably reduced the spread of infectious diseases and could explain reductions in attendances with ‘Infections’.

Increases observed in 0–5 year olds attending with ‘Injuries and Trauma’ during lockdown may reflect challenges in parental supervision of children normally in educational or childcare settings. Conversely, the reduction in 6–18 year olds attending with ‘Injuries and Trauma’ may reflect greater parental confidence in managing injuries in older children and a higher threshold for bringing older children to the ED as compared with babies and pre-school children.

Black African patients were less likely to attend the ED than White British patients in 2020 compared with 2019. Patients reporting Mixed ethnic background were more likely to attend in 2020. Other changes in attendance by ethnicity were minimal, which suggests that changes in healthcare-seeking behaviour during lockdown were not specifically related to ethnicity, though ethnicity data was incomplete.

Using admissions as a proxy for severity at presentation, our analysis suggests that older patients aged ≥ 46 years, males, patients from most Black, Asian and Minority Ethnic groups and from the three most deprived quintiles were more likely to present with more severe clinical disease in 2020 compared with 2019. Possible explanations include more appropriate use of the ED, late presentation and/or hesitancy to access healthcare due to the COVID-19 pandemic. The increased likelihood of admission for these groups was most notable in the ‘Infections’ diagnostic category. It is therefore possible that severity of presentation requiring admission is directly related to COVID-19 infection. Though we were unable to confirm this from the available data, the increased risk of both COVID-19 infection and adverse outcomes from COVID-19 amongst older people, those from Black and Ethnic minority communities, those living in areas of relative deprivation and men has been well described nationally.^11^

Some expected differences were observed in ED attendances by diagnosis in 2020, including an anticipated increase in ‘respiratory infections’ (likely attributable to COVID-19) and a decrease in ‘Injuries and Trauma’ (likely due to the limitations on people’s daily activities during lockdown).

Decreases in attendances were identified across most diagnosis categories. In some instances, non-attendance is unlikely to have led to worse outcomes. For example, the significant drop in attendances for musculoskeletal (non-fracture) diagnoses. Further research to understand and harness this change in health-seeking behaviour may reduce unnecessary ED attendances in the future.

Conversely, there were also reductions in attendances for diagnoses such as ‘acute coronary syndrome/angina’ and ‘stroke’ where prompt access to emergency care is critical for positive clinical outcomes.

### What is already known on this topic

The disruptive impact of COVID-19 on hospital-based and emergency care has been the subject of some studies to date.^12^^,^^13^

National data analyses have found similar shifts in the age distribution of patients attending the ED as observed in this local study.^14^ Whilst the varied reasons for changing patterns in paediatric attendances have not yet been fully explored, a national increase in injuries as a result of abuse and neglect during the pandemic has been described.^15^

Reductions have also been observed nationally in cardiac presentations during the pandemic.^16^

It has been suggested that national media coverage and messaging to ‘Stay home, Protect the NHS, Save lives’ during the first wave was insufficiently nuanced and had an adverse impact on healthcare-seeking behaviours for cardiac and other major conditions requiring emergency care. Further research will be needed to elucidate the impacts of this disruption on potentially avoidable morbidity and mortality.

### What this study adds

This analysis indicates a complex overall picture linked to the interplay between several drivers: the direct impact of COVID-19 infections, changes in NHS service provision during the pandemic, in healthcare-seeking behaviour and in the prevalence of conditions.

In this South East London ED, the direct impact of COVID-19 is demonstrated by findings of a shift towards an older, male, more deprived patient demographic with greater representation of some ethnic minority groups.

The wider impact of the pandemic on healthcare-seeking behaviour is demonstrated with drops in attendances across every demographic characteristic, especially amongst children, and the majority of diagnoses.

The reduction in visits for acute conditions such as stroke and heart attack is concerning. However, it is important to also consider potential positive reductions in attendance due to reduced prevalence of some health conditions, purposeful adaptations of NHS services to keep people out of hospital and appropriate patient choice to avoid seeking emergency care. The pandemic has clearly altered behaviour significantly, and certain behaviour changes may offer an opportunity to achieve a sustained reduction in non-urgent ED visits.

Locally, this analysis may support resource planning and will direct further analysis to understand local health-seeking behaviour change. Understanding patient (and parental) decision-making around when to seek emergency care may help to identify and harness positive behavioural changes and contribute to more appropriate and timely use of ED services in the future. It could also contribute to targeted messaging to encourage ED attendance for those who need it.

### Limitations of this study

Most COVID-19 cases were likely coded as ‘Infection’ however due to its variable profile and lack of testing in the ED, we were unable to quantify COVID-19 specific attendances.

Ethnicity was recorded as ‘unknown’ for a large proportion of attendances in both years, which limits our ability to draw sound conclusions around changes by ethnicity during the pandemic.

We did not consider local disease prevalence and cannot draw conclusions on the health impact of changes in ED attendances for specific diagnoses.

This was a large study conducted at a single major London teaching hospital, serving a relatively young, ethnically and socioeconomically diverse catchment population. Generalisability is limited to similar populations.

## Data sharing

The data underlying this article were provided by King’s College Hospital NHS Foundation Trust by permission. Data will be shared on request to the corresponding author with permission of King’s College Hospital NHS Foundation Trust.

## Supplementary Material

Supplementary_tables_fdab059

## Notes

**Kociejowski A.**, Public Health Specialty Registrar

**Hobart C.**, Public Health Specialty Registrar

**Jina R.**, Senior Clinical Fellow

**Aberman I.**, Medical Student

**Backhurst E.**, Medical Student

**Beaumont A.**, Medical Student

**Crompton J.**, Head of Health Intelligence

**Sneep R.**, Senior Clinical and Research Fellow

**Cantle F.**, Consultant in Adult and Paediatric Emergency Medicine and Major Trauma, Emergency Department Research Lead, Honorary Senior Lecturer Kings College London

**Dodhia H.**, Consultant in Public Health
