# Supplementary material for: Comparison of presentations to the emergency department during the COVID-19 pandemic (COPED-C)
Source: J Public Health (Oxf). 2021 Mar 9;43(4):731–8. doi: 10.1093/pubmed/fdab059 (PMC7989347; doi:10.1093/pubmed/fdab059)
Supplement: Supplementary_tables_fdab059 [file supplementary_tables_fdab059.docx]

## **Supplementary table I: Demographic and Clinical Characteristic Groupings Used for Descriptive Analysis**

| **Age** | | | |
| --- | --- | --- | --- |
| **5 year age groups** | **10 year age groups** | **K-means clustering age groups** | **Condensed Age Groups** |
| 0-4  5-9  10-14  15-19  20-24  25-29  30-34  35-39  40-44  45-49  50-54  55-59  60-64  65-69  70-74  75-79  80-84  85-89  90-94  95-99  100-104  Unknown | 0-9  10-19  20-29  30-39  40-49  50-59  60-69  70-79  80-89  90-99  100-109  Unknown | 0-2  3-5  6-13  14-18  19-33  34-48  49-64  65-78  79+  Unknown | 0-5  6 to 18  19-48  49-64  65+  Unknown |
| **Sex** | | | |
| Male  Female  Both  Unknown | | | |
| **Ethnicity (locally defined categories)** | | | |
| Asian or Asian British  Black or Black British - African  Black or Black British - Any other Black / African / Caribbean background  Black or Black British - Caribbean  Mixed/ multiple ethnic groups  Not known/ not specified  Other ethnic group  Other ethnic group - Latin American  White - any other white background  White - British | | | |
| **Diagnosis** | | | |
| **Full diagnosis categories** | | **Condensed diagnosis categories** | |
| 1. Fractures  2. Musculoskeletal (non-fractures)  3. Wounds & Lacerations  4. Burns  5. Head Trauma  6. Chest/Abdominal Trauma (added to 20. ‘General surgical – emergencies’ due to small numbers  7. Cardiac (except ACS/Unstable Angina)  8. Cardiac ACS/Unstable angina  9. CNS - Emergencies (non-Stroke)  10. CNS - Stroke  11. CNS - Other  12. Diabetic - Emergencies  13. Respiratory - Infections  14. Respiratory emergencies  15. Respiratory - Other  16. GI - Infections & Complications  17. Medical Emergencies - other  18. Soft Tissues & Skin - Infections  19. Soft Tissues - Other  20. General Surgical - Emergencies  21. General Surgical - Other  22. Urological - Emergencies  23. Urological - Infections  24. Urological - Other  25. Gynaecology & Obstetrics - Emergencies  26. Gynaecology & Obstetrics - Infections  27. Gynaecology & Obstetrics - Other  28. Ophthalmology and ENT - Emergencies  29. Ophthalmology and ENT - Infections  30. Ophthalmology and ENT - Other  31. Vascular Emergencies  32. Mental Health (non-substance)  33. Substance Misuse  34. Overdose - Intentional  35. Overdose - Non-intentional (including poisoning) (added to 34. ‘Overdose – Intentional’ due to small numbers)  36. No abnormality detected  37. Vulnerable person (Homeless, safeguarding etc.)  38. Other (Administrative, social, prescribing etc.) | | 1. Injuries & Trauma 2. Medical Emergencies 3. Medical Other 4. Infections 5. Surgical Emergencies 6. Surgical Other 7. Mental Health 8. Overdoses & Poisonings 9. Social/Vulnerable Person 10. Other 11. No abnormality detected | |
| **Outcome** | | | |
| a - Admission (Kings or other)  d - Discharge  r - Died in department  g - Discharged to GP  c - Discharged to Community team (health visitor, school nurse, social worker. mental health etc)  o - Discharged to outpatient department  s - Streamed to other department  u - Ambulatory care unit (medical/surgical)  n - Left before/ after being seen (include triage only)  x - Removed from department  z - Computer or Blank - unknown  h - Occupational Health  m - Missing person | | | |

**Supplementary table II. Study population characteristics: patients admitted to hospital from Emergency department during 2020 and 2019 lockdown weeks (n=24,503)**

|  | **2020** | | **2019** | |  | **95% CI** | |
| --- | --- | --- | --- | --- | --- | --- | --- |
|  | **Number of admissions** | **%** | **Number of admissions** | **%** | **2019-2020**  **% decrease** | **Lower** | **Upper** |
| **Age group** |  |  |  |  |  |  |  |
| *0-5* | 68 | 3.58 | 251 | 7.97 | 72.91 | 66.96 | 78.31 |
| *6-18* | 63 | 3.32 | 238 | 7.56 | 73.53 | 67.44 | 79.02 |
| *19-45* | 392 | 20.64 | 870 | 27.64 | 54.94 | 51.57 | 58.28 |
| *46-64* | 570 | 30.02 | 745 | 23.67 | 23.49 | 20.49 | 26.70 |
| *65-84* | 595 | 31.33 | 748 | 23.76 | 20.45 | 17.62 | 23.53 |
| *≥85* | 211 | 11.11 | 296 | 9.4 | 28.72 | 23.63 | 34.24 |
| *Total* | 1,899 | 100 | 3,148 | 100 | 39.68 | 37.96 | 41.41 |
|  |  |  |  |  |  |  |  |
| **Sex** |  |  |  |  |  |  |  |
| *Male* | 1,084 | 57.02 | 1,623 | 51.54 | 33.21 | 30.92 | 35.56 |
| *Female* | 817 | 42.98 | 1,526 | 48.46 | 46.46 | 43.94 | 49.00 |
| *Total* | 1,901 | 100 | 3,149 | 100 | 39.63 | 37.92 | 41.36 |
|  |  |  |  |  |  |  |  |
| **Ethnic group** |  |  |  |  |  |  |  |
| *Asian* | 97 | 5.1 | 156 | 4.95 | 37.82 | 30.19 | 45.92 |
| *Black African* | 244 | 12.84 | 326 | 10.35 | 25.15 | 20.53 | 30.23 |
| *Black Other* | 174 | 9.15 | 263 | 8.35 | 33.84 | 28.14 | 39.91 |
| *Black Caribbean* | 201 | 10.57 | 276 | 8.76 | 27.17 | 22.01 | 32.83 |
| *Mixed ethnicity* | 36 | 1.89 | 86 | 2.73 | 58.14 | 47.01 | 68.70 |
| *Other ethnic group* | 125 | 6.58 | 214 | 6.8 | 41.59 | 34.91 | 48.50 |
| *Latin American* | 26 | 1.37 | 41 | 1.3 | 36.59 | 22.12 | 53.06 |
| *White British* | 640 | 33.67 | 1,276 | 40.52 | 49.84 | 47.06 | 52.62 |
| *White Other* | 138 | 7.26 | 249 | 7.91 | 44.58 | 38.30 | 50.99 |
| *Unknown/ missing* | 220 | 11.57 | 262 | 8.32 | 16.03 | 11.80 | 21.04 |
| *Total* | 1,901 | 100 | 3,149 | 100 | 39.63 | 37.92 | 41.36 |
|  |  |  |  |  |  |  |  |
| **IMD** |  |  |  |  |  |  |  |
| *1 (Most deprived)* | 512 | 27.48 | 765 | 24.73 | 33.07 | 29.74 | 36.53 |
| *2* | 771 | 41.38 | 1,203 | 38.88 | 35.91 | 33.19 | 38.69 |
| *3* | 408 | 21.9 | 703 | 22.72 | 41.96 | 38.28 | 45.71 |
| *4* | 128 | 6.87 | 294 | 9.5 | 56.46 | 50.58 | 62.21 |
| *5 (Least deprived)* | 44 | 2.36 | 129 | 4.17 | 65.89 | 57.03 | 74.01 |
| ***Total*** | **1,863** | **100** | **3,094** | **100** | **39.79** | **38.06** | **41.54** |

**Supplementary table III. Attendances during 2020 ‘lockdown’ weeks compared to 2019, by diagnosis**

|  | **Number of 1^st^ attendances** | | **Change in attendances (2019 to 2020)** | | |
| --- | --- | --- | --- | --- | --- |
| **Diagnosis Category** | **2019** | **2020** | **% change** | **95% CI** | |
|  |  |  |  | ***Lower*** | ***Upper*** |
| ***Increase in attendances*** |  |  |  |  |  |
| General Surgical – Emergencies (incl. chest/ abdominal trauma) | 39 | 201 | 415.38 | 366.00 | 466.06 |
| Respiratory - Infections | 1408 | 1556 | 10.51 | 8.96 | 12.23 |
| Vulnerable person (Homeless, safeguarding etc) | 15 | 37 | 146.67 | 94.26 | 213.59 |
| ***Decrease in attendances*** |  |  |  |  |  |
| Burns | 124 | 75 | 39.52 | 30.86 | 48.69 |
| Cardiac – Acute coronary syndrome (ACS)/Unstable angina | 153 | 109 | 28.76 | 21.74 | 36.62 |
| Cardiac (except ACS/unstable angina) | 485 | 335 | 30.93 | 26.84 | 35.25 |
| Central Nervous System - other | 739 | 523 | 29.23 | 25.97 | 32.65 |
| Central Nervous System - Stroke | 157 | 140 | 29.82 | 18.43 | 43.40 |
| Central Nervous Systems - Emergencies | 225 | 87 | 61.33 | 54.63 | 67.73 |
| Diabetic Emergencies | 55 | 35 | 36.36 | 23.81 | 50.44 |
| Fractures | 1083 | 418 | 61.40 | 58.43 | 64.31 |
| Gastrointestinal - Infections & Complications | 1910 | 678 | 64.50 | 62.31 | 66.65 |
| General Surgical - Other | 127 | 61 | 51.97 | 42.93 | 60.91 |
| Gynaecology & Obstetrics - Emergencies | 139 | 126 | 9.35 | 5.07 | 15.46 |
| Gynaecology & Obstetrics - Other | 275 | 90 | 67.27 | 61.38 | 72.79 |
| Gynaecology & Obstetrics - Infections | 71 | 31 | 56.34 | 44.05 | 68.09 |
| Head Trauma | 265 | 108 | 59.25 | 53.06 | 65.22 |
| Medical Emergencies - Other | 730 | 1156 | 57.66 | 55.78 | 59.52 |
| Mental Health (non-substance) | 272 | 230 | 15.44 | 11.36 | 20.29 |
| Musculoskeletal (non fractures) | 2409 | 772 | 67.95 | 66.05 | 69.81 |
| No abnormality detected | 1057 | 612 | 42.10 | 39.10 | 45.14 |
| Ophthalmology & ENT - Infections | 770 | 210 | 72.73 | 69.43 | 75.85 |
| Ophthalmology & ENT - Emergencies | 164 | 88 | 46.34 | 38.53 | 54.28 |
| Ophthalmology & ENT - Other | 366 | 176 | 51.91 | 46.66 | 57.13 |
| Other (Administrative, social, prescribing etc.) | 338 | 122 | 63.91 | 58.53 | 69.03 |
| Overdose – Intentional and non-intentional | 112 | 65 | 41.96 | 32.70 | 51.66 |
| Respiratory - other | 45 | 17 | 62.22 | 46.54 | 76.23 |
| Respiratory - Emergencies | 675 | 293 | 56.59 | 52.76 | 60.37 |
| Soft tissues - other | 219 | 144 | 34.25 | 27.99 | 40.94 |
| Soft Tissues & Skin - Infections | 859 | 306 | 64.38 | 61.07 | 67.58 |
| Substance Misuse | 151 | 95 | 37.09 | 29.37 | 45.31 |
| Urological - Emergencies | 156 | 121 | 22.44 | 16.15 | 29.80 |
| Urological - Other | 191 | 122 | 36.13 | 29.32 | 43.37 |
| Urological Infections | 557 | 288 | 48.29 | 44.07 | 52.53 |
| Vascular Emergencies | 219 | 136 | 37.90 | 31.45 | 44.68 |
| Wounds & Lacerations | 2053 | 928 | 54.80 | 52.62 | 56.97 |

**Supplementary table IV. Adjusted Odds Ratio of attendance and admission during 2020 ‘lockdown’ weeks compared to 2019 for top 3 diagnostic categories, by patient characteristics**

**IVa. Injuries and trauma attendances (n=6990) and admissions (n=466)**

|  | **Attendances** | | | |  | **Admissions** | | | |
| --- | --- | --- | --- | --- | --- | --- | --- | --- | --- |
|  |  |  | **95% CI** | |  |  |  | **95% CI** | |
|  | **AOR** | **p value** | **Lower** | **Upper** |  | **AOR** | **p value** | **Lower** | **Upper** |
| **Age (years)** |  |  |  |  |  |  |  |  |  |
| *19-45* | Ref |  |  |  |  | Ref |  |  |  |
| *0-5* | 1.37 | 0.001 | 1.14 | 1.64 |  | 0.48 | 0.15 | 0.18 | 1.30 |
| *6-18* | 0.57 | <0.0001 | 0.48 | 0.68 |  | 0.37 | 0.03 | 0.15 | 0.89 |
| *46-64* | 1.14 | 0.059 | 0.99 | 1.31 |  | 1.46 | 0.24 | 0.78 | 2.74 |
| *65-84* | 1.05 | 0.162 | 0.89 | 2.01 |  | 0.88 | 0.69 | 0.48 | 1.64 |
| *≥85* | 1.34 | 0.162 | 0.89 | 2.01 |  | 1.31 | 0.46 | 0.64 | 2.66 |
|  |  |  |  |  |  |  |  |  |  |
| **Sex** |  |  |  |  |  |  |  |  |  |
| *Female* | Ref |  |  |  |  | Ref |  |  |  |
| *Male* | 0.86 | 0.006 | 0.77 | 0.96 |  | 0.70 | 0.12 | 0.45 | 1.09 |
|  |  |  |  |  |  |  |  |  |  |
| **Ethnicity** |  |  |  |  |  |  |  |  |  |
| *White British* | Ref |  |  |  |  | Ref |  |  |  |
| *Asian* | 0.74 | 0.031 | 0.56 | 0.97 |  | 0.62 | 0.48 | 0.17 | 2.30 |
| *Black African* | 0.67 | <0.0001 | 0.55 | 0.83 |  | 1.05 | 0.91 | 0.46 | 2.41 |
| *Black Caribbean* | 0.67 | 0.001 | 0.53 | 0.84 |  | 1.72 | 0.20 | 0.76 | 3.89 |
| *Black Other* | 0.90 | 0.286 | 0.75 | 1.09 |  | 0.90 | 0.80 | 0.40 | 2.02 |
| *Latin American* | 0.76 | 0.247 | 0.48 | 1.21 |  | 1.66 | 0.45 | 0.45 | 6.19 |
| *Mixed ethnicity* | 1.37 | 0.018 | 1.06 | 1.79 |  | 1.88 | 0.17 | 0.77 | 4.60 |
| *Other ethnic group* | 1.24 | 0.058 | 0.99 | 1.54 |  | 7.37 | 0.12 | 0.58 | 93.55 |
| *White Other* | 0.85 | 0.083 | 0.70 | 1.02 |  | 0.74 | 0.46 | 0.33 | 1.64 |
| *Unknown* | 1.29 | 0.007 | 1.07 | 1.55 |  | 2.19 | 0.04 | 1.03 | 4.64 |
|  |  |  |  |  |  |  |  |  |  |
| **IMD quintile** |  |  |  |  |  |  |  |  |  |
| *Least deprived* | Ref |  |  |  |  | Ref |  |  |  |
| *1 (Most deprived)* | 1.24 | 0.283 | 0.84 | 1.82 |  | 1.00 | 1.00 | 0.30 | 3.31 |
| *2* | 1.23 | 0.288 | 0.84 | 1.79 |  | 0.84 | 0.77 | 0.26 | 2.73 |
| *3* | 1.14 | 0.503 | 0.78 | 1.67 |  | 0.95 | 0.94 | 0.29 | 3.16 |
| *4* | 1.32 | 0.184 | 0.88 | 2.00 |  | 0.59 | 0.44 | 0.15 | 2.26 |

**IVb. Medical emergencies attendances (n= 5,085) and admissions (n=2,431)**

|  | **Attendances** | | | |  | **Admissions** | | | |
| --- | --- | --- | --- | --- | --- | --- | --- | --- | --- |
|  |  |  |  |  |  |  |  |  |  |
|  | **AOR** | **p value** | **95% CI** | |  | **AOR** | **p value** | **95% CI** | |
| **Age (years)** |  |  |  |  |  |  |  |  |  |
| *19-45* | Ref |  |  |  |  | Ref |  |  |  |
| *0-5* | 0.73 | 0.04 | 0.54 | 0.98 |  | 0.72 | 0.17 | 0.45 | 1.15 |
| *6-18* | 0.43 | <0.0001 | 0.32 | 0.59 |  | 0.43 | <0.0001 | 0.26 | 0.73 |
| *46-64* | 1.07 | 0.39 | 0.92 | 1.24 |  | 1.06 | 0.64 | 0.83 | 1.34 |
| *65-84* | 1.13 | 0.17 | 0.95 | 1.34 |  | 1.35 | 0.02 | 1.06 | 1.72 |
| *≥85* | 1.07 | 0.61 | 0.81 | 1.42 |  | 1.18 | 0.36 | 0.83 | 1.67 |
|  |  |  |  |  |  |  |  |  |  |
| **Sex** |  |  |  |  |  |  |  |  |  |
| *Female* | Ref |  |  |  |  | Ref |  |  |  |
| *Male* | 1.11 | 0.09 | 0.98 | 1.25 |  | 1.12 | 0.21 | 0.94 | 1.34 |
|  |  |  |  |  |  |  |  |  |  |
| **Ethnicity** |  |  |  |  |  |  |  |  |  |
| *White British* | Ref |  |  |  |  | Ref |  |  |  |
| *Asian* | 1.02 | 0.91 | 0.77 | 1.35 |  | 1.28 | 0.23 | 0.85 | 1.92 |
| *Black African* | 1.14 | 0.20 | 0.93 | 1.41 |  | 1.36 | 0.04 | 1.01 | 1.84 |
| *Black Caribbean* | 1.11 | 0.36 | 0.89 | 1.39 |  | 1.63 | <0.0001 | 1.18 | 2.27 |
| *Black Other* | 1.28 | 0.03 | 1.02 | 1.59 |  | 1.06 | 0.74 | 0.76 | 1.47 |
| *Latin American* | 0.68 | 0.20 | 0.38 | 1.23 |  | 1.22 | 0.56 | 0.63 | 2.34 |
| *Mixed ethnicity* | 0.93 | 0.74 | 0.60 | 1.43 |  | 0.77 | 0.19 | 0.52 | 1.14 |
| *Other ethnic group* | 0.80 | 0.11 | 0.62 | 1.05 |  | 0.70 | 0.45 | 0.28 | 1.77 |
| *White Other* | 1.32 | 0.02 | 1.04 | 1.67 |  | 1.34 | 0.11 | 0.94 | 1.92 |
| *Unknown* | 1.37 | <0.0001 | 1.11 | 1.69 |  | 1.42 | 0.03 | 1.04 | 1.93 |
|  |  |  |  |  |  |  |  |  |  |
| **IMD quintile** |  |  |  |  |  |  |  |  |  |
| *Least deprived* | Ref |  |  |  |  | Ref |  |  |  |
| *1 (Most deprived)* | 1.23 | 0.24 | 0.87 | 1.73 |  | 1.67 | 0.04 | 1.03 | 2.69 |
| *2* | 1.21 | 0.27 | 0.86 | 1.69 |  | 1.52 | 0.08 | 0.95 | 2.42 |
| *3* | 1.22 | 0.25 | 0.87 | 1.72 |  | 1.47 | 0.12 | 0.91 | 2.39 |
| *4* | 0.85 | 0.42 | 0.58 | 1.25 |  | 1.02 | 0.95 | 0.60 | 1.73 |

**IVc. Infections attendances (n=6,685) and admissions (n=1345)**

|  | **Attendances** | | | |  | **Admissions** | | | |
| --- | --- | --- | --- | --- | --- | --- | --- | --- | --- |
|  | **AOR** | **p value** | **95% CI** | |  | **AOR** | **p value** | **95% CI** | |
| **Age (years)** |  |  |  |  |  |  |  |  |  |
| *19-45* | Ref |  |  |  |  | Ref |  |  |  |
| *0-5* | 0.40 | <0.0001 | 0.34 | 0.48 |  | 0.36 | <0.0001 | 0.20 | 0.67 |
| *6-18* | 0.42 | <0.0001 | 0.34 | 0.52 |  | 0.64 | 0.23 | 0.31 | 1.33 |
| *46-64* | 1.68 | <0.0001 | 1.47 | 1.93 |  | 4.77 | <0.0001 | 3.34 | 6.80 |
| *65-84* | 2.10 | <0.0001 | 1.77 | 2.50 |  | 4.68 | <0.0001 | 3.29 | 6.64 |
| *≥85* | 2.45 | <0.0001 | 1.81 | 3.33 |  | 3.81 | <0.0001 | 2.44 | 5.95 |
|  |  |  |  |  |  |  |  |  |  |
| **Sex** |  |  |  |  |  |  |  |  |  |
| *Female* | Ref |  |  |  |  | Ref |  |  |  |
| *Male* | 1.35 | <0.0001 | 1.22 | 1.51 |  | 1.56 | 0.00 | 1.23 | 2.00 |
|  |  |  |  |  |  |  |  |  |  |
| **Ethnicity** |  |  |  |  |  |  |  |  |  |
| *White British* | Ref |  |  |  |  | Ref |  |  |  |
| *Asian* | 1.04 | 0.77 | 0.82 | 1.31 |  | 1.66 | 0.07 | 0.96 | 2.87 |
| *Black African* | 0.98 | 0.81 | 0.81 | 1.18 |  | 1.92 | <0.0001 | 1.28 | 2.88 |
| *Black Caribbean* | 1.05 | 0.66 | 0.85 | 1.29 |  | 1.62 | 0.03 | 1.04 | 2.53 |
| *Black Other* | 1.05 | 0.60 | 0.87 | 1.28 |  | 2.33 | 0.00 | 1.53 | 3.55 |
| *Latin American* | 1.49 | 0.02 | 1.07 | 2.08 |  | 0.91 | 0.13 | 0.83 | 4.74 |
| *Mixed ethnicity* | 1.20 | 0.24 | 0.88 | 1.63 |  | 2.27 | <0.0001 | 1.35 | 3.81 |
| *Other ethnic group* | 1.37 | 0.01 | 1.10 | 1.70 |  | 1.98 | 0.13 | 0.83 | 4.74 |
| *White Other* | 1.10 | 0.33 | 0.91 | 1.33 |  | 1.29 | 0.32 | 0.79 | 2.11 |
| *Unknown* | 1.78 | <0.0001 | 1.47 | 2.16 |  | 2.50 | <0.0001 | 1.56 | 4.01 |
|  |  |  |  |  |  |  |  |  |  |
| **IMD quintile** |  |  |  |  |  |  |  |  |  |
| *Least deprived* | Ref |  |  |  |  | Ref |  |  |  |
| *1 (Most deprived)* | 0.94 | 0.76 | 0.63 | 1.41 |  | 1.52 | 0.43 | 0.55 | 4.22 |
| *2* | 0.97 | 0.87 | 0.65 | 1.44 |  | 1.49 | 0.44 | 0.54 | 4.09 |
| *3* | 0.94 | 0.75 | 0.62 | 1.40 |  | 1.58 | 0.38 | 0.57 | 4.42 |
| *4* | 0.87 | 0.53 | 0.56 | 1.35 |  | 1.59 | 0.41 | 0.53 | 4.80 |
